# The Throw-and-Catch Model of Human Gait: Evidence from Coupling of Pre-Step Postural Activity and Step Location

**DOI:** 10.3389/fnhum.2016.00635

**Published:** 2016-12-22

**Authors:** Matthew J. Bancroft, Brian L. Day

**Affiliations:** Sobell Department of Motor Neuroscience and Movement Disorders, UCL Institute of Neurology, University College LondonLondon, UK

**Keywords:** postural balance, gait initiation, motor activity, humans, gait, biomechanical phenomena, locomotion, walking

## Abstract

Postural activity normally precedes the lift of a foot from the ground when taking a step, but its function is unclear. The throw-and-catch hypothesis of human gait proposes that the pre-step activity is organized to generate momentum for the body to fall ballistically along a specific trajectory during the step. The trajectory is appropriate for the stepping foot to land at its intended location while at the same time being optimally placed to catch the body and regain balance. The hypothesis therefore predicts a strong coupling between the pre-step activity and step location. Here we examine this coupling when stepping to visually-presented targets at different locations. Ten healthy, young subjects were instructed to step as accurately as possible onto targets placed in five locations that required either different step directions or different step lengths. In 75% of trials, the target location remained constant throughout the step. In the remaining 25% of trials, the intended step location was changed by making the target jump to a new location 96 ms ± 43 ms after initiation of the pre-step activity, long before foot lift. As predicted by the throw-and-catch hypothesis, when the target location remained constant, the pre-step activity led to body momentum at foot lift that was coupled to the intended step location. When the target location jumped, the pre-step activity was adjusted (median latency 223 ms) and prolonged (on average by 69 ms), which altered the body’s momentum at foot lift according to where the target had moved. We conclude that whenever possible the coupling between the pre-step activity and the step location is maintained. This provides further support for the throw-and-catch hypothesis of human gait.

## Introduction

When taking a step, postural activity usually precedes the lift of the stepping foot by around half a second. It modulates the force between the feet and ground to accelerate the body sideways and forwards, and is observed during single steps as well as during locomotion (Carlsöö, 1966; Mann et al., 1979; Crenna and Frigo, 1991; Jian et al., 1993; MacKinnon and Winter, 1993). What is the function of this pre-step activity? One possibility is that its job is to move the body mass directly over the upcoming stance foot, allowing the stepping foot to be lifted freely without compromising balance. However, this is not what is usually observed during single steps or locomotion. At the instant the stepping foot is lifted, the vertical projection of the body’s center of mass (CoM) commonly lies outside and medial to the base of support formed by the stance foot (Jian et al., 1993; MacKinnon and Winter, 1993; Lyon and Day, 1997, 2005). This means that the body is not balanced, but is falling sideways under gravity during a step.

An alternative function of the pre-step activity has been proposed by the throw-and-catch model of human gait (Lyon and Day, 1997). This hypothesis states that the pre-step activity represents a “throw”, which gives the body a specific position and momentum at the time of foot lift. At this point, the body enters a ballistic phase during the step where it falls under gravity along a trajectory determined by the pre-step activity, just as a ball would after being thrown. The direction and magnitude of the throw is finely tuned to take into account the initial state of the body and the intended final position of the stepping foot. Thus, during the step, the stepping foot swings towards its intended target while at the same time being optimally placed when it lands to “catch” the body and regain balance.

The throw-and-catch model predicts that the pre-step activity depends on both the body’s initial conditions and the intended step location. In support of this are the findings that the resulting position and velocity of the body’s CoM at the point of foot lift depend on both the initial stance width (Lyon and Day, 1997) and whether the step is to a forward or diagonal location (Lyon and Day, 1997, 2005), but are not influenced by the final position of the trailing foot (Lyon and Day, 2005). However, the predicted coupling between the pre-step activity and the intended step location has been shown to be breakable under certain circumstances. If the intended step location changes after the stepping foot leaves the ground, for example by shifting the position of a target, it is possible for the foot to land at a location different to that originally planned (Reynolds and Day, 2005, 2007; Kim and Brunt, 2009, 2013; Tseng et al., 2009; Nonnekes et al., 2010). This ability to de-couple the pre-step activity from the step location represents a challenge to the throw-and-catch hypothesis.

Here we investigate the strength of coupling between the pre-step activity and the step location under conditions previously unexplored. First, we study stepping onto five (for each foot) possible target locations, either demanding different step directions with the same step length, or demanding different step lengths with the same direction. The throw-and-catch hypothesis predicts that the pre-step activity will differ and be unique for each target location. Second, we occasionally make the central target jump to another location just after the pre-step activity has been initiated, but before the stepping foot has been lifted. This timing of target jump potentially allows the pre-step activity to be adjusted should it be advantageous to do so. The crucial question, therefore, is whether the pre-step activity is adjusted to take account of the new target location, or is unchanged such that all necessary adjustments are made later during the step, similar to that observed with later target jumps. Support for the throw-and-catch hypothesis would be obtained if: (1) the pre-step activity were modulated by the target jump; and (2) the modulation were dependent on the final location of the target.

## Materials and Methods

Ten human subjects (7 male; 24 ± 2 years; 64 ± 7 kg; mean ± standard deviation (SD)) gave written informed consent to participate in the experiment and reported no known neurological, sensory, muscular or orthopedic disorders. The experiment was approved by the UCL Research Ethics Committee and conformed to the *Declaration of Helsinki*. Participants were provided a written information sheet detailing the experimental procedures. Further to this, the experimental procedures were explained verbally. All subjects were naïve to the purpose of the study.

### Protocol

**Figure 1:**
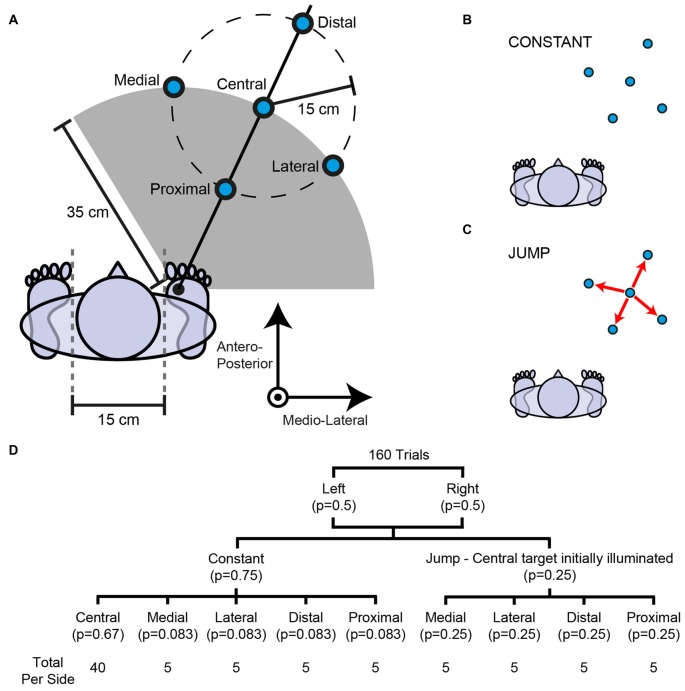
**Experimental set-up. (A)** Right-sided targets are depicted; an identical configuration was present on the left side. The vertical axis protrudes directly out of the page. Subjects were instructed to step to a visually-presented target (blue circles). In most trials a target illuminated on either the left or right side and its location did not change **(B)**. In some trials the target jumped 15 cm from the central target to one of four peripheral targets (medial, lateral, distal or proximal) shortly after initiation of the pre-step activity **(C)**. **(D)** Probability tree outlining the number of trials and probability per condition. A target jump occurred with a probability of 0.33 after illumination of the central target.

Subjects performed a step onto a floor-bound target. Prior to the step, subjects stood barefoot and still with both feet parallel. The medial borders of the feet were separated by 15 cm. This starting position was chalk-marked to ensure a consistent starting location for all steps. The positions of the targets required a forwards movement, with five targets located on each of the left and right sides (Figure 1). Subjects were instructed to step as accurately as possible to the target, and bring the trailing foot alongside. An accurate step to each target was self-defined by all participants prior to the experiment. To achieve this, participants placed the appropriate foot over each of the 10 targets without time constraints in a manner they deemed accurate. For these accuracy trials, subjects were allowed to adjust their foot’s position until they felt it represented an accurate step to a target. The step was redone if the subject or experimenter felt it necessary. Subjects were instructed to land their foot in this way for all subsequent steps and the position of their foot when it landed was compared to this ideal position. By instructing subjects to step accurately to the targets, rather than as quickly as possible, we sought to minimize variability in the placement of the stepping foot and thereby minimize variability in the step location.

The final position of the stepping foot was emphasized as being paramount and non-adjustable following foot landing. After the stepping foot landed subjects were required to step with the trailing foot and bring it alongside their stepping foot. The final position of the trailing foot does not affect the performance of the initial step (Lyon and Day, 2005) so no specific instruction was given as to where to place the trailing foot. However, subjects were encouraged to finish the step in a balanced state, similar to which they started. When a target illuminated to the left, a left-foot leading step was required and when a target illuminated to the right, a right-foot leading step was required. Multiple targets on both left and right sides were used to prevent prediction of target jump location or stepping side, thereby ensuring unbiased conditions at the time of target presentation and target jump.

A trial began with an audible beep, which was followed by a random delay and illumination of a step target. Illumination of the target acted as a cue for the subject to initiate a step to its location in their own time. The stepping targets were oriented so that step length and direction could be independently manipulated (Figure 1A). That is, for medial, central and lateral targets, step length was constant (35 cm) but step direction was different (0°, 25°, 50° forward of the stepping foot respectively). Equally, for proximal, central and distal targets, step direction was constant (25° forward of the stepping foot) but step length was different (20 cm, 35 cm, 50 cm respectively).

### Experimental Conditions

In 75% of trials, one of the five targets on either the left or right side was selected pseudo-randomly and illuminated for the duration of the step (constant condition; Figure 1B). The central target was selected most frequently (probability = 0.67), whereas the peripheral targets were selected less often but each with equal frequency (probability = 0.083).

In 25% of trials, the central target was illuminated on either the left or right side and was made to unpredictably jump to one of its four peripheral targets selected pseudo-randomly (jump condition; Figure 1C). The peripheral targets were selected with equal frequency when a target jumped to a new location (probability = 0.25). After illumination of the central target, the probability of it jumping was 0.33.

The target jump was achieved by simultaneously extinguishing the central target and illuminating one of the four peripheral targets. Target jumps were triggered when the difference in vertical force between the stepping and trailing foot sides exceeded 80 N. This related to a mean (SD) of 7.8 (0.9)% (range: 5.7%–8.8%) of total body weight. Due to inherent electrical transmission delays, the new target would emit light 16.5 ms after the trigger signal. This delay is accounted for in all reported data. The new target appeared shortly after the initiation of the pre-step activity (mean (SD) latency: 96 (43) ms), long before the stepping foot lifted. The locations of the new targets meant that the required magnitude of foot adjustment remained constant (15 cm) but either step direction (medial- or lateral-jump) or length (distal- or proximal-jump) required modification.

A total of 160 trials were completed and performed in 10 blocks of 16 steps. The number of trials and its probability for each condition is summarized in Figure 1D.

### Apparatus

Targets were circular (2.5 cm diameter) and illuminated via electroluminescent paper (Light Tape UK Limited, Barnsley, Yorkshire, UK) within a low-profile display on the floor in front of the subject. Ambient light conditions were dim (<0.1 Lux; Isotech 1332A Digital Illuminance Meter, Southport, Merseyside, UK) to eliminate potential distractors and ensure that the target light was compelling. Three infrared-emitting diode markers were placed at the base of the first metatarsal and head of the first (hallux) and fifth metatarsals of each foot. Marker positions were tracked at 200 Hz by two motion capture units comprising six “cameras” (Codamotion cx-1, Charnwood Dynamics, Leicestershire, UK). At the start of a trial, subjects stood with each foot over separate force platforms (9281C1, Kistler, Winterthur, Switzerland) which were embedded in the floor. Force was acquired at 1000 Hz.

### Data Analysis

Both marker position and force data were digitally low-pass filtered using a zero-lag, second order Butterworth filter with 15 Hz and 30 Hz cut-off frequencies respectively. An anti-aliasing analog filter was used on force data prior to this.

The two force platforms were summed to evaluate the net force acting on the body in three-dimensions. From Newton’s Second Law of Motion, the acceleration of the body’s CoM was calculated by dividing net force by a subject’s mass. The net acceleration of the body’s CoM while the subject stood still (from the beginning of a trial until target illumination) was assumed to be zero and used as a baseline for the remainder of the trial. CoM velocity at foot lift, which has previously been shown to be an important variable in stepping (Lyon and Day, 1997, 2005), was then estimated by numerical integration of the CoM acceleration during the pre-step activity (trapezoidal method).

Typically, the center of pressure initially moves laterally towards the stepping side heel during the pre-step activity (Mann et al., 1979; Breniere et al., 1987). Therefore, the pre-step activity’s initiation was calculated as the first point after target illumination that the medio-lateral (ML) center-of-pressure velocity towards the stepping foot exceeded 5 cm.s^−1^ for at least 50 ms. Stepping foot lift was the first point that the stepping side’s vertical force went below 1% of total body weight. Pre-step activity duration was the time from its initiation to stepping foot lift. The time the stepping foot landed was the first point after foot lift that the ML, antero-posterior (AP) and vertical speed of the stepping foot hallux marker went below 2 cm.s^−1^. Speed was calculated as the absolute value of the first derivative of marker position. The final position of the stepping foot was the mean position of the three foot markers upon landing.

**Figure 2:**
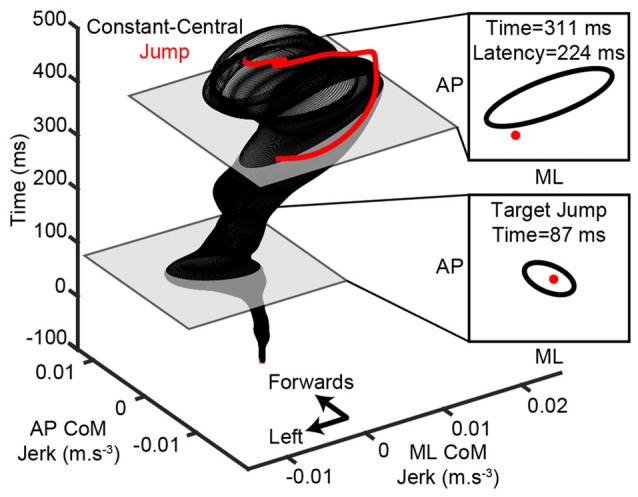
**Calculation of modulation latency.** An example is shown for one subject’s step to the right-side lateral target after the target jumped (red). Medio-lateral (ML) and antero-posterior (AP) center-of-mass (CoM) jerk traces were aligned to the initiation of the pre-step activity (time = 0) and 95% confidence ellipses were calculated at each time point for the constant-central steps (black). A modulation was detected when a jump trial diverged from the confidence ellipse. In this example the modulation occurred 224 ms after the target jumped and before the stepping foot lifted (time = 488).

To investigate whether the pre-step activity was modulated when the intended step location changed, the body’s horizontal (AP and ML) motion in jump trials was compared to constant-central (the initial target in a jump trial) steps over time after alignment. All trials were aligned to the initiation of the pre-step activity and CoM acceleration was differentiated to calculate jerk. Two-dimensional 95% confidence intervals (confidence ellipses) were then generated at each time point for the constant-central condition. Figure 2 shows an example of this for one subject. The latency of any modulation was defined as the first time, at least 100 ms after a target jump, that a jump trial diverged from the constant-central confidence interval. The delay of 100 ms after a target jump was chosen as this relates to the shortest reported latencies to adjust lower limb trajectory or modulate ground reaction forces in visuomotor tasks (Reynolds and Day, 2005, 2007; Leonard et al., 2011). Modulation latency was measured for each jump condition and subject.

Sixteen jump trials (1% of total trials, 4% of jump trials) were excluded from the analysis due to the target jump being triggered either before the initiation of the pre-step activity or shortly before the stepping foot lifted.

### Statistical Analysis

No bias was found between the left and right sides or dominant and non-dominant legs in the latency of the target jump from the pre-step activity’s initiation, final position of the stepping foot, CoM velocity at foot lift, or duration of the pre-step activity (all *P* > 0.05). Therefore left-sided steps were reflected about the laboratory AP axis and combined with right-sided steps. All steps are reported as if they were right-sided.

Foot placement and CoM velocity at foot lift contained both ML and AP components and as such are multidimensional variables requiring analysis by multidimensional statistical methods. Ideally, a repeated-measures ANOVA capable of analyzing differences in multidimensional variables would be used to test whether the foot landed accurately or whether CoM velocity differed with target location. However, to the best of our knowledge, no such test exists. Therefore, paired *t*-tests for multidimensional variables (one-sample Hotelling’s tests; Batschelet, 1981; Zar, 2010) were used to distinguish differences between conditions. Using multidimensional statistical methods, rather than examining the data in two dimensions separately, is advantageous as power is increased (Batschelet, 1981) and no *a priori* assumptions are required about which dimension of the data an effect is expected. CoM velocity at foot lift was paired within-subject by subtracting the mean of the constant-central condition from all other stepping conditions.

Unidimensional temporal variables (pre-step activity duration and modulation latency) were submitted to repeated-measures ANOVA with the within-subject factor of step location. ANOVAs were performed using SPSS statistical software (IBM Corporation, New York, NY, USA). Hotelling’s tests and all other analyses were performed using custom written Matlab scripts (The Mathworks Inc., Natick, MA, USA). Statistical significance was set at an alpha level of 0.05 after Bonferroni correction for multiple comparisons. Greenhouse Geisser correction was used in ANOVAs when the assumption of sphericity was violated. Normality of data analyzed by Hotelling’s tests was confirmed by Mardia’s test of skewness and kurtosis (Mardia, 1970).

## Results

### Steps When the Target Location Remained Constant

**Figure 3:**
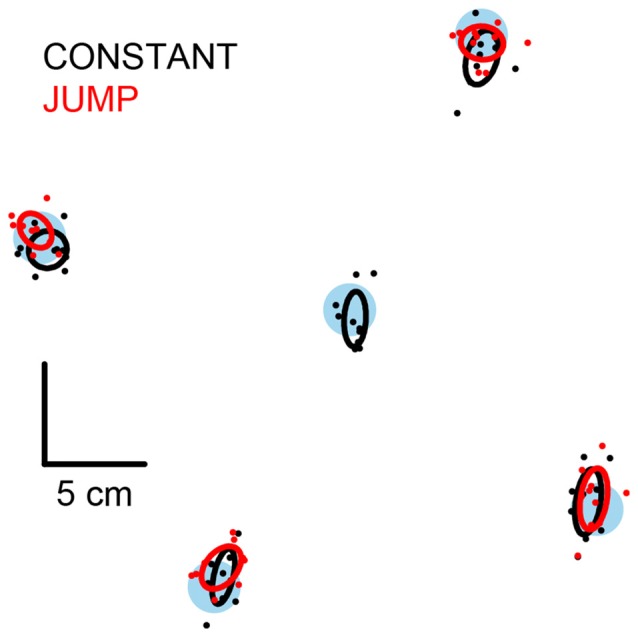
**Mean final position of the stepping foot.** Filled blue circles are the stepping targets. Filled black and red circles are each subject’s mean final position of the stepping foot for the constant and jump conditions respectively. Ellipses are Hotelling’s 95% confidence ellipses of the group mean. The size and spacing of the stepping targets is to scale.

All subjects were able to land the stepping foot on or near the target when its location remained constant. On average, the final position of the foot was not different to each subject’s self-defined ideal step, which was the case when stepping to all target locations (Figure 3, black symbols; all *T*^2^_(2,8)_ < 5.3, *P* > 0.7). This indicated that a successful step was taken to all targets.

**Figure 4:**
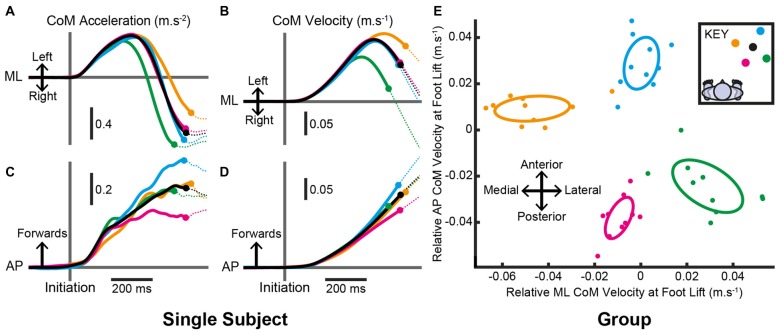
**Centre-of-mass (CoM) motion when the target location remained constant. (A–D)** Mean estimated CoM acceleration **(A,C)** and velocity **(B,D)** over time from an individual subject in the ML **(A,B)** and AP **(C,D)** dimensions. Trials were aligned to the time of pre-step activity initiation. Filled circles denote the mean time the stepping foot lifted in steps to each target and the traces are dotted thereafter. **(E)** Estimated CoM velocity at foot lift for the group after within-subject pairing by subtracting the constant-central mean. Filled circles are each subject’s mean estimated CoM velocity at foot lift for each target. Ellipses are Hotelling’s 95% confidence ellipses of the group mean. As per the key, black= central; yellow= medial; green= lateral; blue= distal; magenta= proximal.

In order to land the foot in this position, all subjects produced pre-step activity that accelerated the body sideways, away from the stepping foot, and forwards. The mean (SD) time from the pre-step activity’s initiation until foot lift was 561 (89) ms and was not consistently affected by the step location (*F*_(4,36)_ = 0.8, *P* = 0.520). However, the pre-step activity that accelerated the CoM differed with target location (Figure 4). Figures 4A,C depict the mean CoM acceleration for one subject and shows activity before the lift of the stepping foot. Figures 4B,D show that for this subject the pre-step acceleration resulted in the body gaining velocity that was specific for each intended step location. For the group analysis, each subject’s mean CoM velocity at foot lift for steps to the central target was subtracted from the mean value for steps to each peripheral target. The resulting two-dimensional (AP and ML) representation of relative CoM velocity at foot lift showed a target-specific organization that resembled the relative positions of the targets in Cartesian coordinates (Figure 4E). The confidence ellipses of CoM velocity at foot lift for steps to medial, lateral, distal and proximal locations were all significantly different from each other (all *T*^2^_(2,8)_ > 82.5, *P* < 0.001), indicating that the pre-step activity was coupled to the planned step location.

### Steps When the Target Location Changed

All subjects adjusted their step when the target jumped to a new position shortly after initiation of the pre-step activity. This was demonstrated by the final position of the foot being different to that originally planned (constant-central steps) for steps to all target-jump locations (Figure 3, red symbols; all *T*^2^_(2,8)_ > 1900, *P* < 0.001). Furthermore, the mean final position of the foot was not different to each subject’s self-defined ideal step for any target jump location (all *T*^2^_(2,8)_ < 8.0, *P* > 0.3), indicating that the step adjustments were successful and accurate. The final position of the foot after a target jump was mostly the same as when stepping without a target jump (lateral, distal and proximal all *T*^2^_(2,8)_ < 10.5, *P* > 0.18), however medial steps were modestly but significantly different from each other (mean vector distance = 1.1 cm; *T*^2^_(2,8)_ = 49.2, *P* = 0.002).

**Figure 5:**
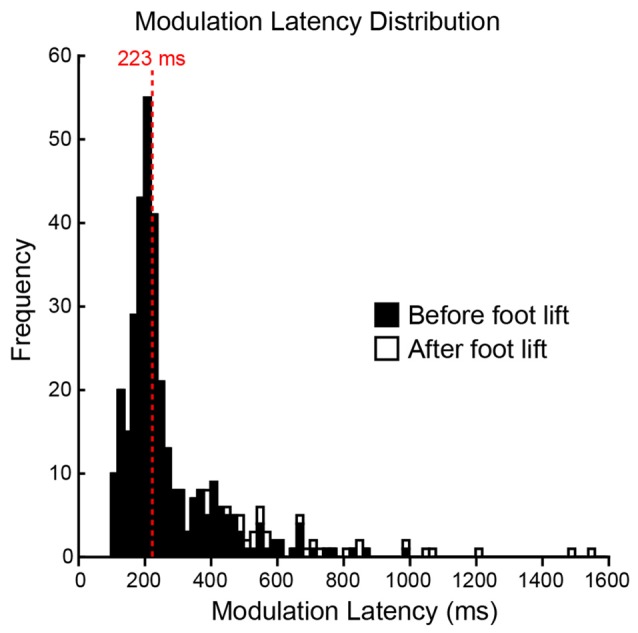
**Distribution of modulation latencies.** A histogram of all jump trials for all subjects is depicted. The distribution was skewed and the average modulation latency was determined by the median (223 ms, red dashed line). Filled bars represent modulations occurring before the lift of the stepping foot and open bars represent modulations occurring after the lift of the stepping foot. Each bin is 20 ms.

To determine whether the pre-step activity was modulated in response to a change in step location, the CoM motion when subjects stepped to the constant-central target was compared with that when the target jumped (see Figure 2 for details). An initial modulation could be reliably detected in 94% of all jump trials, with 6% of trials being excluded because their jerk trace lay outside the constant-central ellipse at the time of target jump. The first detectable modulation occurred before the foot lifted from the ground in the vast majority (93%) of the remaining trials, with a modulation after foot lift in 7% of trials (Figure 5). The median modulation latency was 223 ms (interquartile range = 158) and was not affected by the new target’s location (*F*_(3,27)_ = 1.0, *P* = 0.41). Subjects would also delay the lift of the stepping foot to elongate the pre-step activity in response to the change of target location. When compared with steps to the constant-central target, foot lift was significantly delayed by 69 (55) ms in jump trials (*P* = 0.003), but did not depend on target-jump location (*F*_(3,27)_ = 1.8, *P* = 0.178). The stepping foot was also lifted later in jump trials than in steps to the same location without a target jump (*F*_(1,9)_ = 10.9, *P* = 0.009). The mean (SD) pre-step activity duration of steps with a target jump was 629 (129) ms.

**Figure 6:**
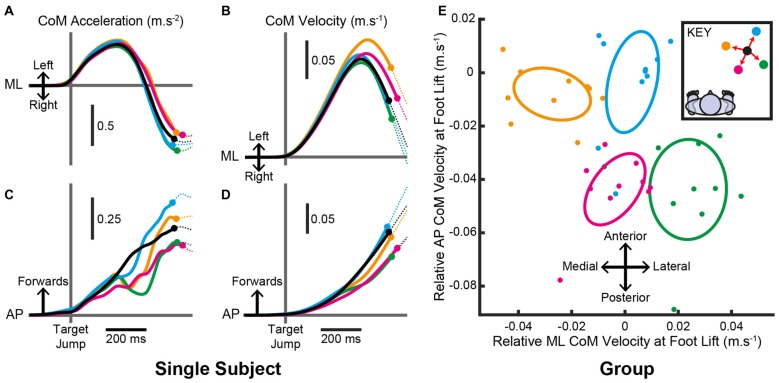
**Centre-of-mass (CoM) motion when the target location changed. (A–D)** Mean estimated CoM acceleration **(A,C)** and velocity **(B,D)** over time from an individual subject in the ML **(A,B)** and AP **(C,D)** dimensions. Trials were aligned to the time a target jumped. Filled circles denote the mean time the stepping foot lifted in steps to each target and the traces are dotted thereafter. **(E)** Estimated CoM velocity at foot lift for the group after within-subject pairing by subtracting the constant-central mean. Filled circles are each subject’s mean estimated CoM velocity at foot lift for each target. Ellipses are Hotelling’s 95% confidence ellipses of the group mean. As per the key, black = constant-central; yellow = medial; green = lateral; blue = distal; magenta = proximal.

Typically, the initial response to a change in target location acted to reduce the forward acceleration of the body, as shown for a single subject in Figure 6C. This “braking” effect was apparent for all target jump locations even if an increase in forward acceleration seemed more appropriate, for example during a jump that required an increased step length. The initial non-specific response was rapidly followed by a target-specific acceleration of the CoM (Figures 6A,C) leading to different CoM velocities at the point of foot lift (Figures 6B,D). For the group, the confidence ellipses of the change in CoM velocity at foot lift for steps with a medial, lateral, distal and proximal target jump were all significantly different from each other (Figure 6E; all *T*^2^_(2,8)_ > 83.9, *P* < 0.001) demonstrating that the pre-step activity modulation was target-specific. The modulated CoM velocity at foot lift reflected the positions of the new stepping target, similar to that observed when the target position remained constant, suggesting that an attempt was made to re-couple the pre-step activity and step location.

## Discussion

The experiments were designed to test the throw-and-catch hypothesis of human gait. This was achieved by measuring the coupling between the pre-step activity (the throw) and the final stepping-foot position (the catch). The hypothesis states that the two actions are intimately coupled such that the pre-step activity differs with the planned step location. This was verified in the experiment where the final location was known to the subject throughout the movement. For the experiment in which the final location changed just after the throw was initiated, which effectively de-coupled the two actions, the throw was found to be adjusted so that it was re-coupled to the new step location. Together these results provide support for the throw-and-catch hypothesis of human gait.

### Steps When the Target Location Remained Constant

The throw-and-catch model predicts that the pre-step activity depends on the intended step location. This is because the direction and magnitude of the body throw would need to be tuned differently in order for the body to fall towards its target during steps to different locations. Previous research identified a coupling between the pre-step activity and step location for forwards and diagonal steps (Lyon and Day, 1997, 2005). We sought to investigate whether this finding, and the throw-and-catch model, generalized to steps of different lengths and directions. The resulting data therefore required that both ML and AP body motion was analyzed together using multidimensional statistics (Hotelling’s tests and confidence ellipses). The results confirmed that the pre-step activity systematically differed with target location in steps of both different lengths and directions (Figure 4). The net result of these changes was such that when the stepping foot lifted from the ground, the body had gained velocity, and therefore momentum, that was specific to the intended step location. Lyon and Day (1997) demonstrated that the body’s momentum at the point of foot lift is a key factor that predicts the body’s trajectory during the step. The coupling between the body’s momentum and step location in the present results suggests that this ballistic model generalizes and the throw is fine-tuned for steps of different lengths and directions.

In order to test the throw-and-catch model’s generality, steps were initiated from an imposed initial posture which allowed both step length and direction to be precisely controlled. Although the initial posture may not have been a natural stance width or foot angle for all subjects, it is unlikely to have affected their behavior significantly. The step was otherwise natural for each subject as the initiation and duration of the step was unconstrained. This lack of temporal constraint is unlikely to have affected the use of a ballistic strategy, as it has been demonstrated both for temporally unconstrained steps (Lyon and Day, 1997, 2005) and when initiating gait as quickly as possible (Yiou et al., 2016). It is possible that differences in step duration may have influenced the throws to different targets (Zettel et al., 2002a,b; Yiou et al., 2016). However, this is unlikely to explain our data because although step duration changed with step length, it did not change with step direction (data not shown). In exploring the throw-and-catch in steps of different lengths and directions, subjects would often step beyond the force platforms. Subsequently, CoM position was unable to be reliably estimated (for a discussion of CoM position estimation from force data see Lyon and Day, 1997). The position from which the body initiates its fall is an important aspect of the throw (Lyon and Day, 1997, 2005) but, by demonstrating a step location-specific change in CoM velocity, the present results show that the pre-step activity is tuned to the future foot position.

The between-subject variability of the throw in this experiment (Figure 4E) and in Lyon and Day (1997) shows that there is no “one-size-fits-all” throw to achieve a successful step. Even for the same subject, different throws could successfully be caught by identical foot-landing positions. This likely reflects both the ability to make mid-step adjustments (Reynolds and Day, 2005, 2007; Kim and Brunt, 2009, 2013; Tseng et al., 2009; Nonnekes et al., 2010) and the numerous solutions that exist to catch the body without losing balance (Koolen et al., 2012). However, the present results show that there is pressure to maintain the throw within limits which differ for different step locations. This suggests there is some advantage in coupling the pre-step activity and step location.

### Steps When the Target Location Changed

The pre-step activity and the step location can be uncoupled, for example by changing the location of a target. In this scenario the body throw and step location become uncoupled if the pre-step activity remains unchanged while the foot position changes. Previous work has shown that if the target is made to jump after the foot leaves the ground, so that the pre-step activity cannot be changed, the final position of the stepping foot can still be altered, albeit with varying degrees of success depending on the extent and direction of the target jump (Reynolds and Day, 2005, 2007; Kim and Brunt, 2009, 2013; Tseng et al., 2009; Nonnekes et al., 2010). This de-coupling does not necessarily disprove the throw-and-catch hypothesis, but may be interpreted as there being some flexibility in the coupling which can be exploited under certain circumstances. A better test of the hypothesis is to measure whether there is an attempt to re-couple the pre-step activity with the new step location under conditions when the pre-step activity has an opportunity to be adjusted. Here we made the target change location during the pre-step activity, long before the stepping foot left the ground, thus allowing time for a re-coupling of the pre-step activity with the new target location. The target jump was unpredictable, as more often than not the target location did not change and could be to any one of four locations when it did. This procedure resulted in substantial uncertainty and rendered attempts at anticipating the target jump unlikely to be of value to the subject. We argue that an attempt to re-couple the pre-step activity with the new step location would provide support for the throw-and-catch hypothesis given that an adjustment could be initiated after the foot leaves the ground.

Previous research is conflicted on whether the pre-step activity can be modulated. Experiments that unpredictably perturbed the trajectory of the body during the pre-step activity via mechanical pulls have returned mixed results, with muscle activity being altered to correct the body’s trajectory in response to resistive (Mouchnino et al., 2012; Mille et al., 2014) but not assistive perturbations (Mouchnino et al., 2012). Additionally, stimulation of proprioceptive (Ruget et al., 2008) and vestibular (Bent et al., 2002) afferents were found not to affect the pre-step activity, suggesting it may be immutable once it has been initiated. This was not found to be the case in the present experiment, since the pre-step activity was modulated in the vast majority of trials when prompted by a visual cue. Furthermore, the precise modulation of the pre-step activity depended on the new target location. This suggests that an attempt was made to re-couple the pre-step activity to the new target location and provides causal evidence that the pre-step activity is fine-tuned to take the final position of the foot into account. According to the throw-and-catch hypothesis, re-coupling the pre-step activity to the new target location would have promoted a more appropriate fall of the body towards its intended target after the lift of the stepping foot. This does not rule out the possibility that further adjustments were made during the step.

Although the net adjustment of the pre-step activity was different depending on where the target jumped, a non-specific initial response to the perturbation was evident that reduced the forward acceleration of the body. Similar “braking” responses to visual cues have been reported after the lift of the stepping foot during both single steps and locomotion (Kim and Brunt, 2013; Potocanac et al., 2016). In the present experiment, this braking was used irrespective of where the target moved. Although the braking could have been expected when the step length required shortening, it was surprising that it was also used when a longer step was required. This is because greater forward acceleration was observed for longer steps when the target did not move (Figure 4). The braking may have enabled a pause whilst the necessary actions for a successful step were reconsidered (Potocanac et al., 2016), which would explain the increase in pre-step duration that was observed when a target jumped. Mille et al. (2014) also report a similar delay in stepping after a mechanical pull to the body prior to a step. Presumably, in the current experiments, the extra time was needed to re-program and adjust the body throw so that it was re-coupled to the new final foot location.

### What Advantage Does the Throw-and-Catch Coupling Offer?

Stepping can be considered a ballistic action if, as we suggest, gravity drives the fall of the body during the step with its trajectory being controlled by the pre-step activity. The ballistic nature of stepping is sometimes misinterpreted as sensory information not being attended to or usable during the step. This interpretation is clearly false given that adjustments to the step are possible both before the lift of the stepping foot, as shown in this paper, and after foot lift when suitable sensory cues are provided (Reynolds and Day, 2005, 2007; Kim and Brunt, 2009, 2013; Tseng et al., 2009; Nonnekes et al., 2010). However, the magnitude and direction of mid-step adjustments are limited by the fall of the body and the subsequent balance constraints. For example, the size of medially-directed foot adjustments cannot be made as large as laterally-directed adjustments (Reynolds and Day, 2005). This limitation may underlie the incentive to maintain the coupling between the pre-step activity and final foot location demonstrated in the present experiment. At the same time, the ability to rapidly alter the intended foot position mid-step offers a degree of flexibility in the coupling, which would be essential for responding to an unexpected environmental threat or maintaining foot landing accuracy in the presence of error in the initial throw.

## Conclusion

We find that there is a close coupling between pre-step activity and final foot position during unperturbed steps of different lengths and directions. Furthermore, there is pressure to maintain this coupling when the demanded step location unpredictably changes shortly after initiation of the pre-step activity. We conclude that these results support the throw-and-catch hypothesis of human stepping.

## Author Contributions

MJB and BLD conceptualized and designed the experiment, analyzed the data, drafted and revised the manuscript and approved its final version. MJB acquired the data.

## Funding

This work was funded by a University College London (UCL) Grand Challenge studentship.

## Conflict of Interest Statement

The authors declare that the research was conducted in the absence of any commercial or financial relationships that could be construed as a potential conflict of interest.
